# Supplementary material for: Causal Relationship between Aging and Anorexia Nervosa: A White-Matter-Microstructure-Mediated Mendelian Randomization Analysis
Source: Biomedicines. 2024 Aug 16;12(8):1874. doi: 10.3390/biomedicines12081874 (PMC11351342; doi:10.3390/biomedicines12081874)
Supplement: Supplementary file 1 [file biomedicines-12-01874-s001.zip › Supplementary Figure/Figure S4/Singe SNP plot of MR analysis for TL to ubm-b-1533.pdf]

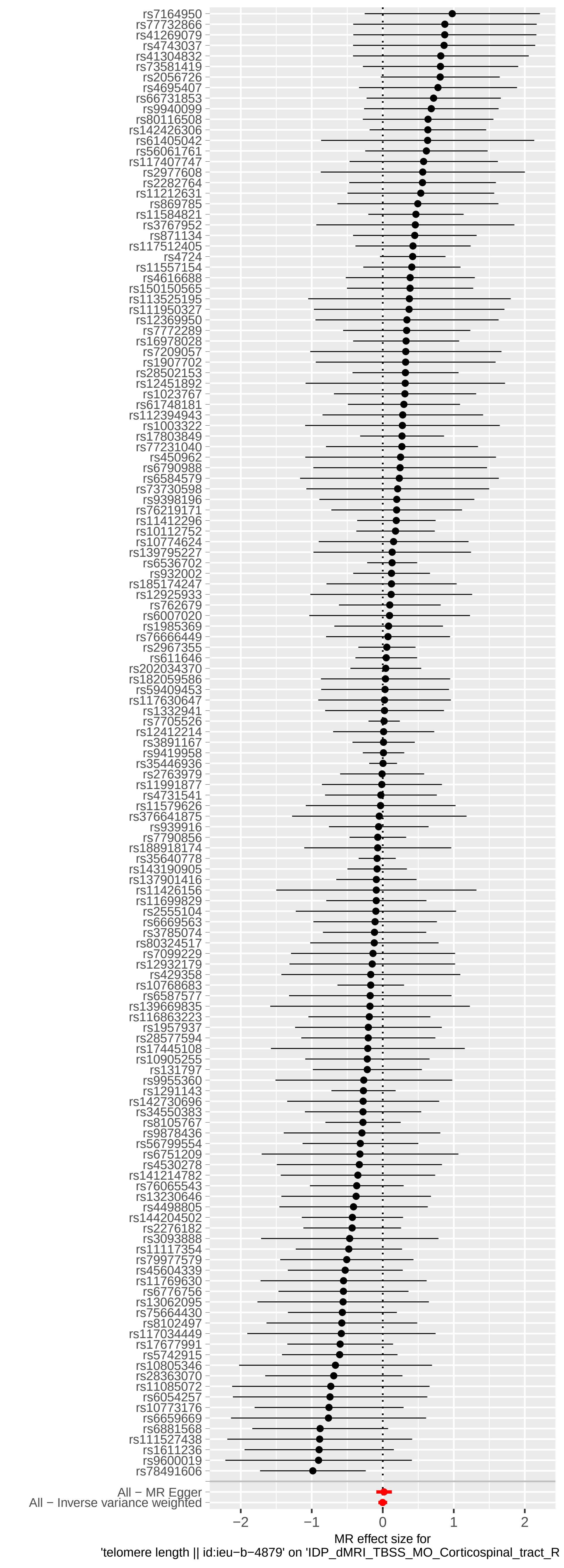
rs7164950
rs77732866
rs41269079
rs4743037
rs41304832
rs73581419
rs2056726
rs4695407
rs66731853
rs9940099
rs80116508
rs142426306
rs61405042
rs56061761
rs117407747
rs2977608
rs2282764
rs11212631
rs869785
rs11584821
rs3767952
rs871134
rs117512405
rs4724
rs11557154
rs4616688
rs150150565
rs113525195
rs111950327
rs12369950
rs7772289
rs16978028
rs7209057
rs1907702
rs28502153
rs12451892
rs1023767
rs61748181
rs112394943
rs1003322
rs17803849
rs77231040
rs450962
rs6790988
rs6584579
rs73730598
rs9398196
rs76219171
rs11412296
rs10112752
rs10774624
rs139795227
rs6536702
rs932002
rs185174247
rs12925933
rs762679
rs6007020
rs1985369
rs76666449
rs2967355
rs611646
rs202034370
rs182059586
rs59409453
rs117630647
rs1332941
rs7705526
rs12412214
rs3891167
rs9419958
rs35446936
rs2763979
rs11991877
rs4731541
rs11579626
rs376641875
rs939916
rs7790856
rs188918174
rs35640778
rs143190905
rs137901416
rs11426156
rs11699829
rs2555104
rs6669563
rs3785074
rs80324517
rs7099229
rs12932179
rs429358
rs10768683
rs6587577
rs139669835
rs116863223
rs1957937
rs28577594
rs17445108
rs10905255
rs131797
rs9955360
rs1291143
rs142730696
rs34550383
rs8105767
rs9878436
rs56799554
rs6751209
rs4530278
rs141214782
rs76065543
rs13230646
rs4498805
rs144204502
rs2276182
rs3093888
rs11117354
rs79977579
rs45604339
rs11769630
rs6776756
rs13062095
rs75664430
rs8102497
rs117034449
rs17677991
rs5742915
rs10805346
rs28363070
rs11085072
rs6054257
rs10773176
rs6659669
rs6881568
rs111527438
rs1611236
rs9600019
rs78491606
All − MR Egger
All − Inverse variance weighted
−2
−1
0
1
2
MR effect size for
'telomere length || id:ieu−b−4879' on 'IDP_dMRI_TBSS_MO_Corticospinal_tract_R
